# A muscle growth-promoting treatment based on the attenuation of activin/myostatin signalling results in long-term testicular abnormalities

**DOI:** 10.1242/dmm.047555

**Published:** 2021-02-19

**Authors:** Danielle Vaughan, Robert Mitchell, Oliver Kretz, David Chambers, Maciej Lalowski, Helge Amthor, Olli Ritvos, Arja Pasternack, Antonios Matsakas, Sakthivel Vaiyapuri, Tobias B. Huber, Bernd Denecke, Abir Mukherjee, Darius Widera, Ketan Patel

**Affiliations:** 1 School of Biological Sciences, University of Reading, Reading NW1 0TU, UK; 2 III. Department of Medicine, University Medical Center Hamburg-Eppendorf, Hamburg 20251, Germany; 3 Functional Genomics, King's College London, London SE1 1UL, UK; 4 Department of Biochemistry and Developmental Biology, HiLIFE, Meilahti Clinical Proteomics Core Facility, University of Helsinki, Helsinki 00014, Finland; 5 Versailles Saint-Quentin-en-Yvelines University, INSERM U1179, LIA BAHN CSM, Montigny-le-Bretonneux 78180, France; 6 Department of Bacteriology and Immunology, University of Helsinki, Helsinki 00014, Finland; 7 Molecular Physiology Laboratory, Centre for Atherothrombosis and Metabolic Disease, Hull York Medical School, Hull HU6 7RX, UK; 8 School of Pharmacy, University of Reading, Reading RG6 6UB, UK; 9 RWRTH Aachen University, Aachen 52062, Germany; 10 Royal Veterinary College, London NW1 0TU, UK

**Keywords:** Activin, Gene array, Muscle, Myostatin, Testis

## Abstract

Activin/myostatin signalling acts to induce skeletal muscle atrophy in adult mammals by inhibiting protein synthesis as well as promoting protein and organelle turnover. Numerous strategies have been successfully developed to attenuate the signalling properties of these molecules, which result in augmenting muscle growth. However, these molecules, in particular activin, play major roles in tissue homeostasis in numerous organs of the mammalian body. We have recently shown that although the attenuation of activin/myostatin results in robust muscle growth, it also has a detrimental impact on the testis. Here, we aimed to discover the long-term consequences of a brief period of exposure to muscle growth-promoting molecules in the testis. We demonstrate that muscle hypertrophy promoted by a soluble activin type IIB ligand trap (sActRIIB) is a short-lived phenomenon. In stark contrast, short-term treatment with sActRIIB results in immediate impact on the testis, which persists after the sessions of the intervention. Gene array analysis identified an expansion in aberrant gene expression over time in the testis, initiated by a brief exposure to muscle growth-promoting molecules. The impact on the testis results in decreased organ size as well as quantitative and qualitative impact on sperm. Finally, we have used a drug-repurposing strategy to exploit the gene expression data to identify a compound – *N*^6^-methyladenosine – that may protect the testis from the impact of the muscle growth-promoting regime. This work indicates the potential long-term harmful effects of strategies aimed at promoting muscle growth by attenuating activin/myostatin signalling. Furthermore, we have identified a molecule that could, in the future, be used to overcome the detrimental impact of sActRIIB treatment on the testis.

## Abstract (precis)

**Editor’s choice:** Brief exposure of the mouse testis to a muscle growth-promoting molecule initiates a cascade of molecular changes that expands over time, even when the treatment has stopped.

## INTRODUCTION

Skeletal muscle is the largest organ found in the human body, in which it can account for ∼50% of the entire mass. As well as facilitating movement, this organ performs a variety of additional roles, including protecting internal organs, maintaining body posture, preserving constant body temperature, and assisting movement in cardiovascular and lymphatic vessels. Skeletal muscle is a highly adaptable organ that changes not only its mass but also its composition to meet the physiological needs of the body (Matsakas and Patel, 2009). Furthermore, skeletal muscle undergoes a huge degree of maintenance to sustain its functional capabilities through a programme of organelle surveillance and protein turnover. It has been estimated that an average adult weighing 70 kg has a daily protein turnover of ∼420 g per day, a vast amount of which takes place in skeletal muscle (Waterlow and Jackson, 1981; Slevin et al., 1991). Huge advances have been made in our molecular understanding of the mechanisms that control muscle mass, which have identified three key processes (protein synthesis, protein breakdown and autophagy) that act to keep this organ in an optimal functional state, which involve balancing hypertrophic and atrophy-promoting activities (Sandri et al., 2004).

Given that skeletal muscle performs so many vital functions, it is hardly surprising that diseases manifest when mechanisms that control its homeostasis become imbalanced. Perturbations can be induced by genetic changes in molecules that control muscle structure and function. One of the best studied mediators of this class of change is Duchenne muscular dystrophy (DMD), caused by mutations in the dystrophin gene, which encodes a molecule that acts to prevent contraction-mediated cell damage. However, it is likely that much of the population will experience issues related to muscle through the process of sarcopenia, age-related muscle loss. This is a very significant problem especially in the developing world, where there is an unprecedented increase in the proportion of elderly people. Sarcopenia increases both morbidity and mortality rates (Sobestiansky et al., 2019) and puts a tremendous burden on the economy. The hospitalisation-associated costs amount to over $19 billion per year in the USA alone (Goates et al., 2019). Given these facts, it is understandable why great efforts were made to develop therapies to treat muscle-wasting conditions.

We and others have focused on manipulating the activin/myostatin signalling pathway to promote growth, following the discovery that these molecules are potent inhibitors of skeletal muscle hyperplasia and hypertrophy (McPherron et al., 1997; Lee and McPherron, 2001; Matsakas et al., 2009; Alyodawi et al., 2019). This axis is an attractive therapeutic target as the ligands mediating muscle growth inhibition are secreted by cells, thereby raising the possibility for systemic route interventions. Several approaches have been successfully developed that attenuate activin/myostatin signalling and lead to robust muscle growth in adult mammalian experimental models. These include the use of activin/myostatin-binding proteins, such as follistatin, and receptor-blocking antibodies (Amthor et al., 2004; Lach-Trifilieff et al., 2014).

As an alternative, we have developed an activin/myostatin ligand trap (sActRIIB), which comprises the extracellular portion of the activin type IIB receptor (ActRIIB; also known as ACVR2B), as well as modifications that promote peptide stability. In a series of studies, we have shown that this molecule induces robust muscle growth in a matter of a few weeks when systemically administered into mice irrespective of their age, disease state or metabolic condition (Relizani et al., 2014; Alyodawi et al., 2019; Omairi et al., 2016). The utility of these molecules as therapies for muscle-wasting conditions, however, needs to be carefully scrutinised, because the activin/myostatin signalling pathways are deployed for the homeostasis of numerous organs and sActRIIB has broad ligand-binding properties, with ligands including members of the bone morphogenetic protein (BMP) family (Greenwald et al., 2003). This line of thinking has been supported in primate studies, which reported sActRIIB-induced changes in the spleen and pancreas and glucose homeostasis (Latres et al., 2017).

Recently, we have shown that, while promoting muscle growth, sActRIIB attenuated the development of the testis (Vaughan et al., 2020a,b). In these studies, we showed that, soon after its administration, sActRIIB inhibited testis development and function when it was injected into mice at any stage of their post-natal life, as well as inhibited testis development in two mice models of DMD. Importantly, the long-term consequence of sActRIIB on muscle and testis development remained unexplored.

In this study, we aimed to determine the molecular mechanisms underpinning the testis phenotype induced by sActRIIB treatment, as well as utilise a bioinformatics-based approach to identify readily available compounds that would mitigate this phenotype. We hypothesised that the effect of a sActRIIB regime on testicular molecular signature would be short term and reversible after the end of the intervention, resembling the effects on skeletal muscle. We used a microarray-based approach to develop a transcriptomic profile of the testis to investigate the short- and long-term sActRIIB action. Our results show that sActRIIB only promotes short-lived muscle growth, in contrast to a persistent testicular phenotype, which at the molecular level inflates over time. Molecular changes induced by sActRIIB were predicted to impact on microtubule function, which was verified by ultrastructural examination of sperm. Finally, Connectivity Map (CMAP)-based drug repurposing (Lamb et al., 2006) identified one molecule that would potentially attenuate the testis phenotype in a temporal-independent manner.

## RESULTS

**Fig. 1.**
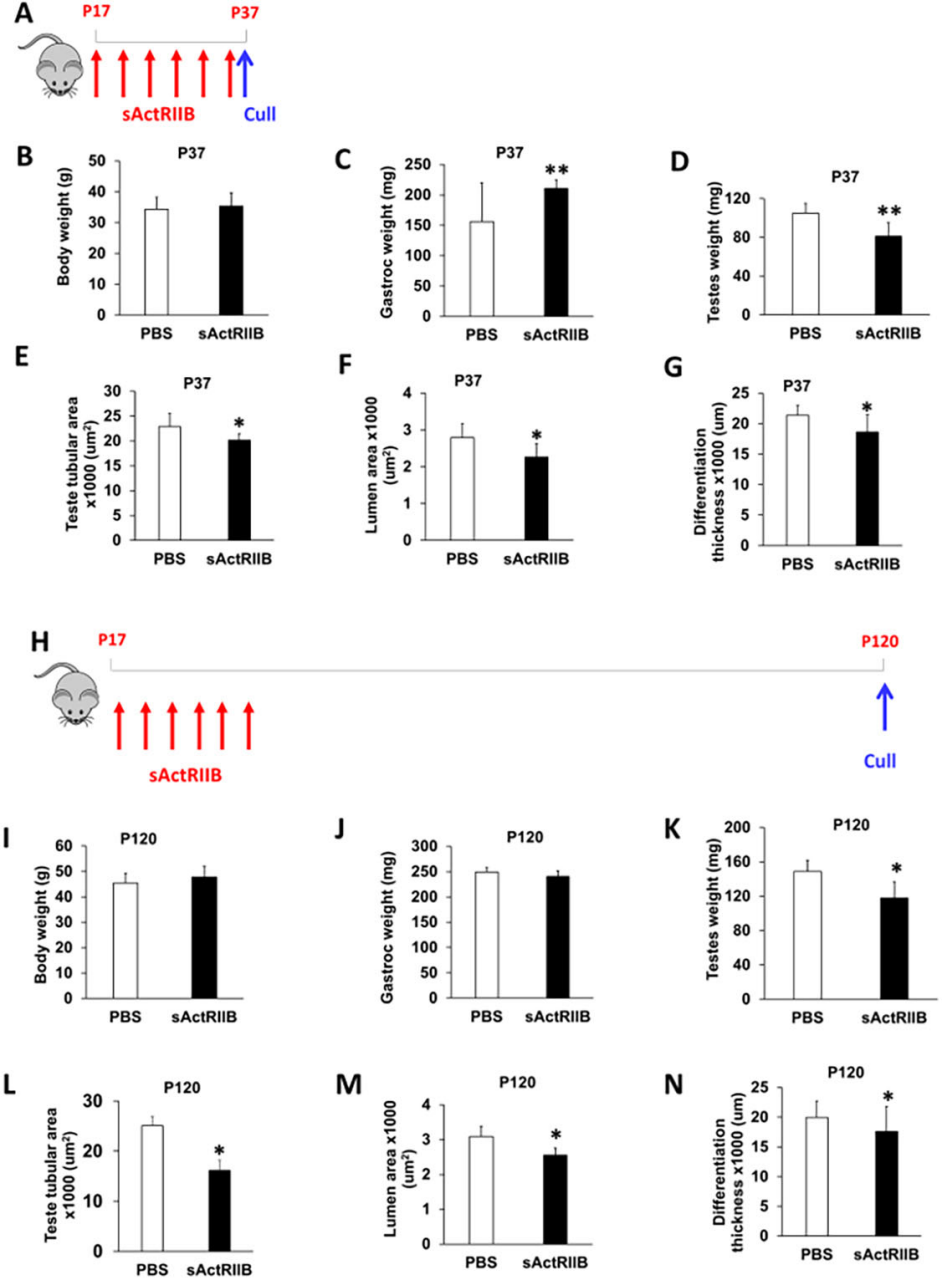
**sActRIIB causes a reduction in testicular development in mice at P37 and P120.** (A) Schematic of experimental design for P37 treatment. Red arrows indicate injection of 10 mg/kg sActRIIB; blue arrow indicates time of cull. (B-G) For P37, body weight (B), gastrocnemius mass (C), testis mass (D), average tubule area (E), average lumen area (F) and average differentiation thickness (G) in the PBS- and sActRIIB-treated mice. *n*=7 PBS-treated male mice, *n*=7 sActRIIB-treated male mice. (H) Schematic of experimental design for P120 treatment. Red arrows indicate injection of 10 mg/kg sActRIIB; blue arrow indicates time of cull. (I-N) For P120, body weight (I), gastrocnemius mass (J), testis mass (K), average tubule area (L), average lumen area (M) and average differentiation thickness (N) in the PBS- and sActRIIB-treated mice. *n*=4 PBS-treated male mice, *n*=4 sActRIIB-treated male mice. Unpaired Student's *t*-test was used to determine statistical significance. *P<0.05, **P<0.01.

We have previously shown that sActRIIB promoted robust muscle growth in several mouse models (Alyodawi et al., 2019). In keeping with those results, we here showed that a short treatment (∼20 days) induced muscle growth, without any significant changes in body weight (Fig. 1A-C; Fig. S1A-D). However, even this short treatment caused a decrease in testis weight (Fig. 1D). Examination of histological sections of testis from mouse treated at post-natal day (P)17 to P37 revealed that the seminiferous tubular area was reduced, as was the differentiation thickness and the tubule lumen area (Fig. 1E-G). We then investigated whether the reduction in testis weight in adult mice caused by sActRIIB treatment of pre-pubertal mice was of a permanent nature or whether this effect was reversible. To that end, we injected mice until P35 and examined tissues at P120 (Fig. 1H). Interestingly, at this time point, there was no difference in body weight or muscle mass between the two cohorts (Fig. 1I,J). However, the testes of sActRIIB-treated mice were lighter than those of controls (Fig. 1K), and three metrics of testicular development (tubule area, lumen area, differentiation thickness) were reduced (Fig. 1L-N). We next analysed whether the decrease in tubule area, lumen area and differentiation thickness was a read out of a decrease in testis weight. Therefore, we calculated the ratio of these parameters normalised to the weight of the testis and then compared the ratios for tissues from control mice to those from treated animals. Interestingly, the decrease in the measures of tubule area, lumen area and differentiation thickness appears to reflect the decrease in testis size (Fig. S1E-J). However, the ratio between lumen area and testis weight at P120 was significantly different between control and sActRIIB-treated mice, indicating an effect of the sActRIIB treatment (Fig. S1H). Hence, most, but not all, internal decreases in tissue architecture can be explained by the smaller size of the overall testis.

**Fig. 2.**
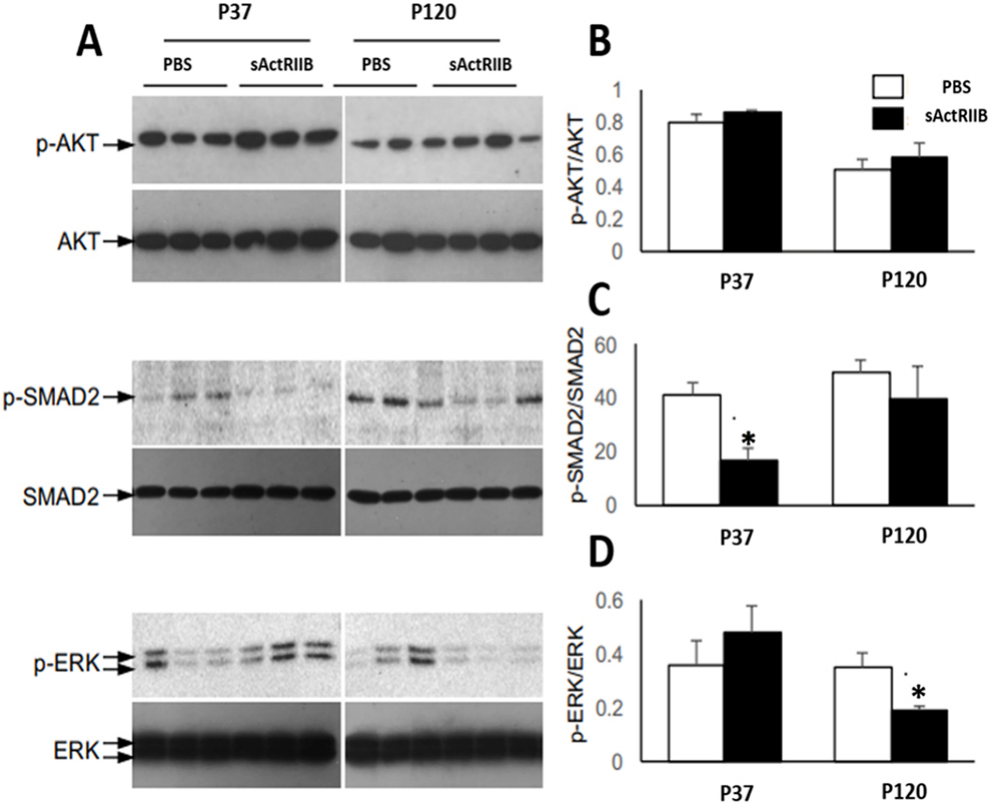
**Altered signalling in testis upon sActRIIB treatment.** (A) Immunoblot analyses of testis lysates from P35 (young) or P120 (adult) mice treated with PBS or sActRIIB (see Materials and Methods for treatment times) for either phosphorylated (p) or total target proteins (AKT, SMAD2 and ERK) as indicated. (B-D) Bar graphs representing densitometric quantification of phosphorylated target protein normalised to total target protein from the western blotting experiments shown in A: pAKT/AKT (B), pSMAD2/SMAD2 (C) and pERK/ERK (D). Unpaired Student's *t*-test was used to determine statistical significance. *n*=3, *P<0.05.

We next investigated the possible causes for the change in testis size following sActRIIB treatment by examining key signalling pathways thought to be impacted by activin/myostatin signalling. To test whether sActRIIB treatment effectively reduces activin signalling in the testis, we performed western blotting with protein lysates prepared from P37 and P120 mice treated with either sActRIIB or PBS. As shown in Fig. 2, there was a significant reduction in the ratio of phospho (p)SMAD2/SMAD2 (the most proximal signalling effect after ActR engagement) in testes from sActRIIB-treated mice at P37, but this effect does not persist at P120 (Fig. 2A,C). This, therefore, suggests that the proximal effects of TGFβ signalling inhibition might be the cause of spermatogenic defects as discussed above. The AKT signalling pathway was shown to be induced in a model of increased activin activity, in which the testis size is increased (Oldknow et al., 2013). Hence, we investigated whether, in our model of reduced activin action and reduced testicular size, AKT activation is reduced. Interestingly, in our studies, we did not find any reduction in AKT activity, suggesting that AKT signalling might not play an important role in the testes of mice treated with sActRIIB (Fig. 2A,B). We failed to identify any significant changes in pERK/ERK (also known as MAPK) at the early time point, but they were reduced by treatment at P120 (Fig. 2A,D).

**Fig. 3.**
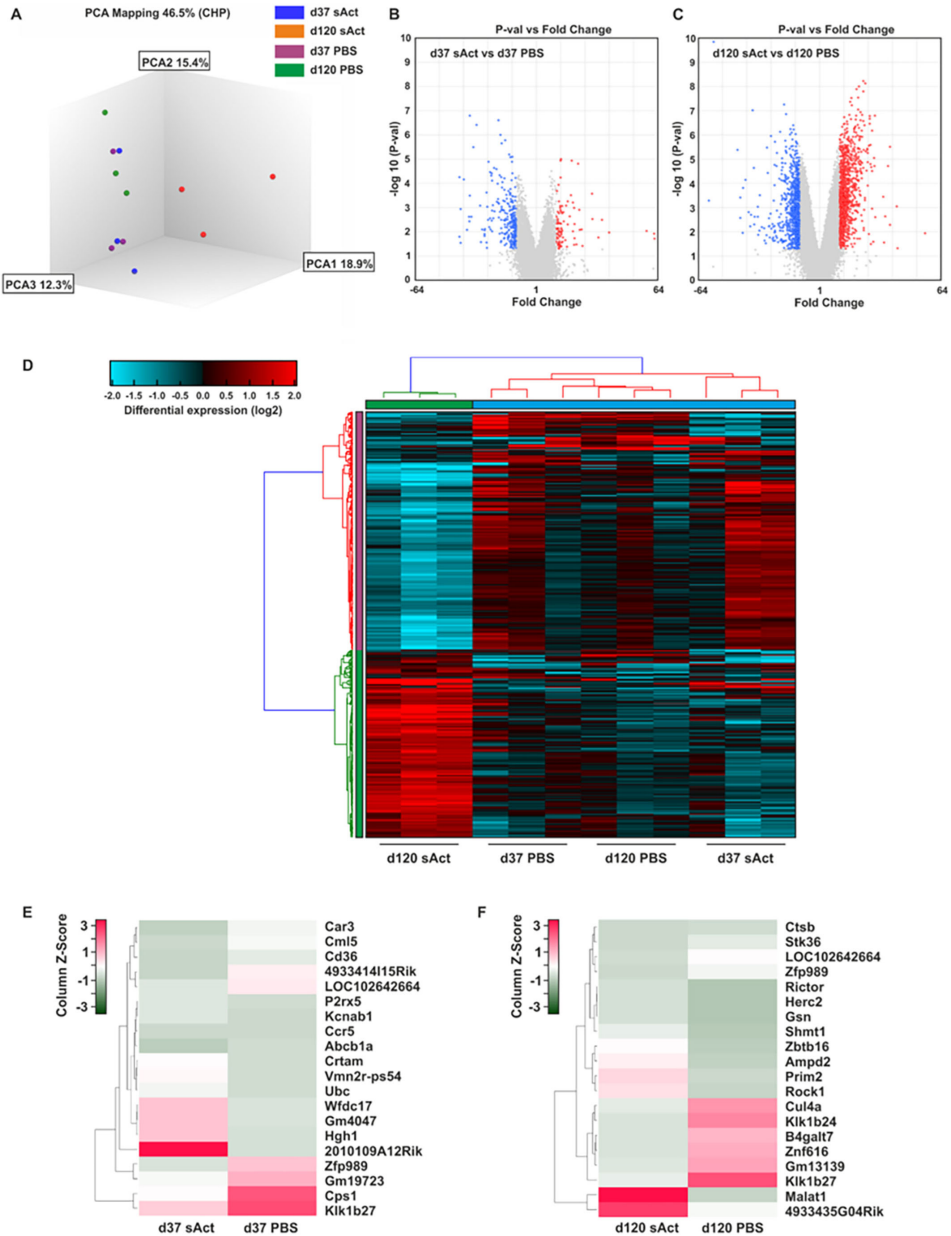
**Bioinformatic comparison of the gene expression in sActRIIB-treated mice and control animals.** (A) Principal component 3D-analysis (PCA) of the gene expression data set. Each dot represents a sample. (B,C) Volcano plot analysis showing log2 fold change and *P*-value of each transcript. Each gene is represented by a dot on the graph. The *x*-axis represents the log2 value of fold change, and the *y*-axis represents the t-statistic as log10 *P*-value. Coloured dots represent the genes that are ≥2-fold upregulated (red) or downregulated (blue) with a *P*-value lower than 0.05. In total, 355 genes were found to be differentially expressed in sActRIIB-treated animals and controls at P37 (B), and 1801 genes were found to be differentially expressed at P120 (C). (D) Hierarchical clustering. Each column represents one individual animal. The colour and intensity of the boxes are used to represent changes (not absolute values) in gene expression. Red represents upregulated genes; blue represents downregulated genes; black represents unchanged expression. Only genes significantly (*P*<0.05) differentially expressed with a 3-fold minimal change in expression are illustrated. (E,F) Heat map representations of differentially expressed transcripts at P37 (E) and P120 (F), with the highest degree of change sorted according to *z*-score ranking. Euclidean distance measures and average leaf clustering were applied to rows. The list of differentially altered transcripts with the highest degree of change of expression in each condition is shown in the column on the right (corresponding gene symbols). The colours correspond to the *z*-score, ranging from green (low expression) to red (high expression).

To identify the differential genome-wide gene expression patterns potentially responsible for the changes in testis size, microarray analyses from micro-dissected testes of sActRIIB-treated and control animals were performed. Analysis of the microarray by principal component analysis (Fig. 3A) revealed differences in genome-wide gene expression patterns between the sActRIIB-treated and control animals at P37 that became more prominent at P120, suggesting a strong impact of the sActRIIB injection. Notably, gene expression in the testes of control animals at P37 and P120 clustered closer with that of the sActRIIB-treated animals at P37 than with that of the sActRIIB-treated animals at P120. Overall, we detected 355 differentially expressed genes between sActRIIB-treated and control animals at P37 (98 upregulated and 257 downregulated genes), whereas 1801 genes showed a differential expression pattern at P120 (1003 upregulated and 798 downregulated genes). Volcano plots comparing gene expression between control and sActRIIB-treated mice further emphasised that the gene expression patterns exhibit more differences at P120 than at P37 (Fig. 3B,C). Importantly, as evidenced by the heat map in Fig. 3D, at both time points, the arrays for each experimental group yielded similar data. A distinct gene expression pattern was observed, differentiating the sActRIIB-treated and control animals at both time points (Fig. 3D).

A more in-depth examination into the most differentially expressed genes between sActRIIB-treated mice and control animals at P37 revealed strong downregulation of multiple genes including *Car3*, *Cml5* (also known as *Nat8f5*) and *Cd36*, but strong upregulation of *Wfdc17*, *Gm4047* and *Hgh1*, in sActRIIB-treated mice (Fig. 3E). Consistent with the principal component analysis (PCA), the differences in gene expression at P120 were more pronounced and revealed strong upregulation of *Malat1*, but strong downregulation of *Klk1b24* and *Klk1b27*, in animals treated with sActRIIB (Fig. 3F).

**Fig. 4.**
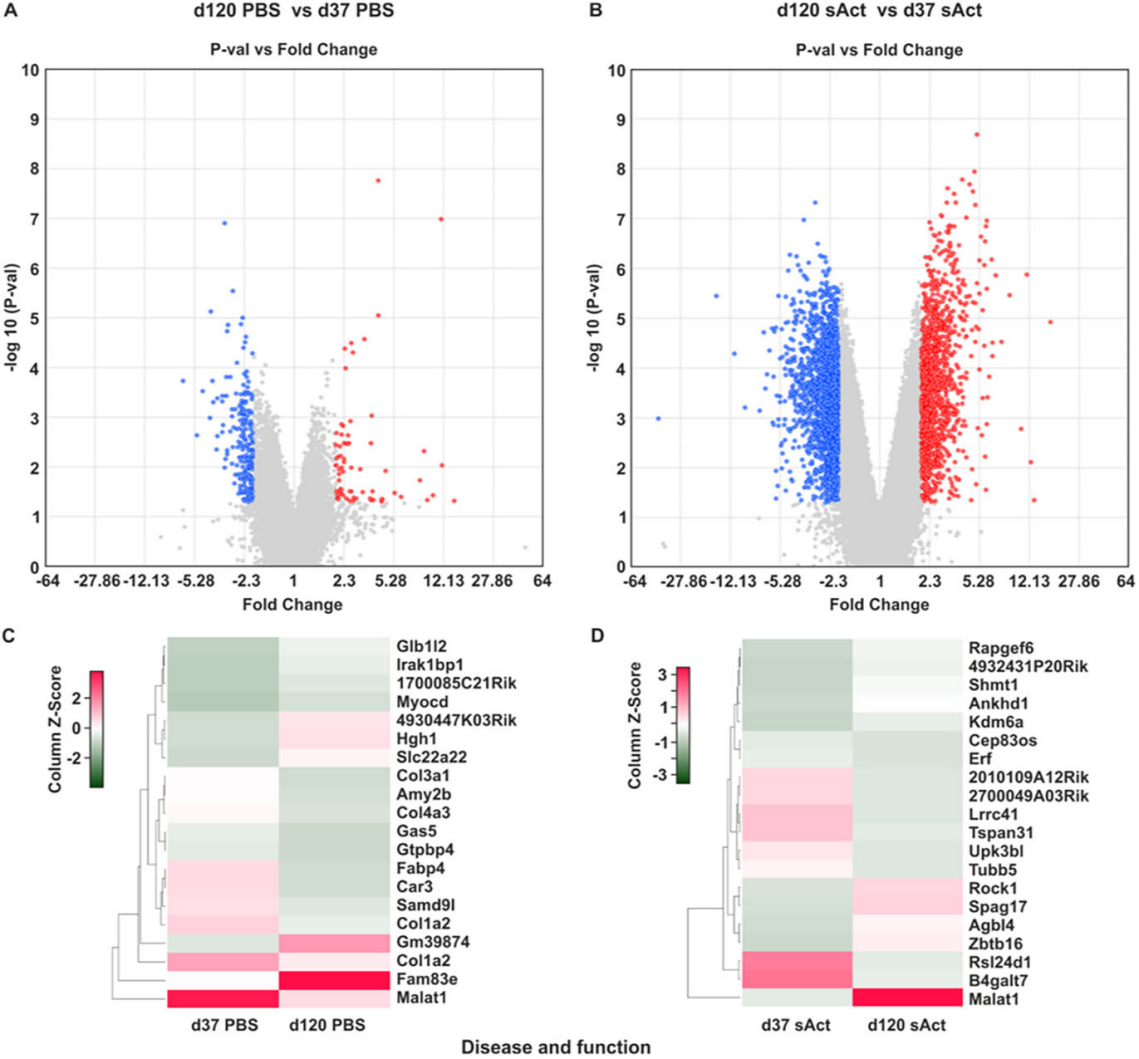
**Bioinformatic comparison of testis gene expression in sActRIIB-treated mice.** (A,B) Volcano plot analysis showing log2 fold change and *P*-value of each transcript at P120 versus P37 in PBS-treated mice (A) and sActRIIB-treated mice (B). The *x*-axis represents the log2 value of fold change, and the *y*-axis represents the t-statistic as log10 *P*-value. Coloured dots represent the genes that are ≥2-fold up- or downregulated with a *P*-value lower than 0.05. Upregulated and downregulated genes are highlighted in red and blue, respectively. (C,D) Heat map representations of differentially expressed transcripts with the highest degree of change, sorted according to *z*-score ranking, in PBS-treated mice (C) and sActRIIB-treated mice (D). Euclidean distance measures and average leaf clustering were applied to rows. The list of differentially altered transcripts with the highest degree of change of expression in a given condition is shown in the column on the right (corresponding gene symbols).

Volcano plot analysis of the differential expression pattern at P37 versus P120 in control animals showed less prominent differences. Notably, more pronounced differences were observed when directly comparing the expression pattern in testes of animal treated with sActRIIB at both respective time points (Fig. 4A,B). An in-depth analysis showed strong downregulation of *Malat1* at P120 compared to P37 in the control group. In contrast, *Malat1* was upregulated at P120 compared to P37 in the sActRIIB-treated group (Fig. 4C,D).

Next, in order to identify potential biological processes and canonical pathways responsible for the differences between the experimental groups, we performed an analysis of down- and upregulated canonical pathways and pathways involved in disease and specific cellular functions using the Ingenuity Pathway Analysis (IPA) tool (Figs S2 and S3). Here, we demonstrated strong downregulation of multiple signalling pathways involved in cell proliferation in the sActRIIB group at P37, including NF-κB and IL-6 signalling. In contrast, IPA revealed strong upregulation of the negative regulator of proliferation (Lu et al., 2016) PTEN compared to the control group at P37 (Figs S2 and S3).

**Fig. 5.**
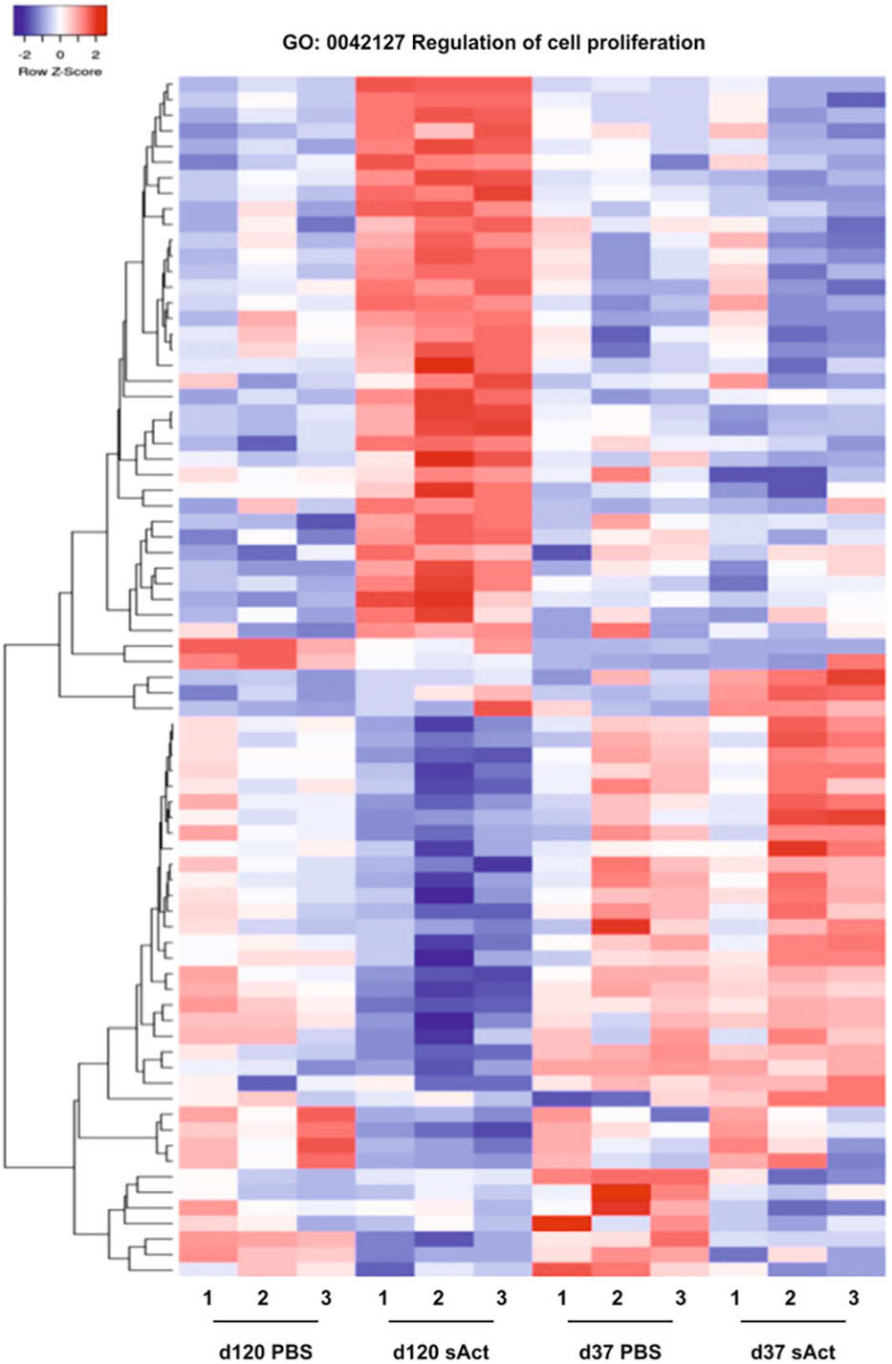
**Heat map representations of differentially expressed transcripts within the gene ontology category ‘Regulation of cell proliferation’.** Euclidean distance measures and average leaf clustering were applied to rows. The experimental conditions are indicated on the bottom. Each column represents one individual animal.

As IPA revealed an impact of sActRIIB on several processes and signalling pathways linked to cell proliferation, an in-depth analysis of genes linked to cell proliferation was performed. A heat map analysis of the respective gene profiles revealed profound differences between the gene expression patterns of control and sActRIIB-treated animals at P120, whereas less prominent effects were revealed at P37 (Fig. 5).

**Fig. 6.**
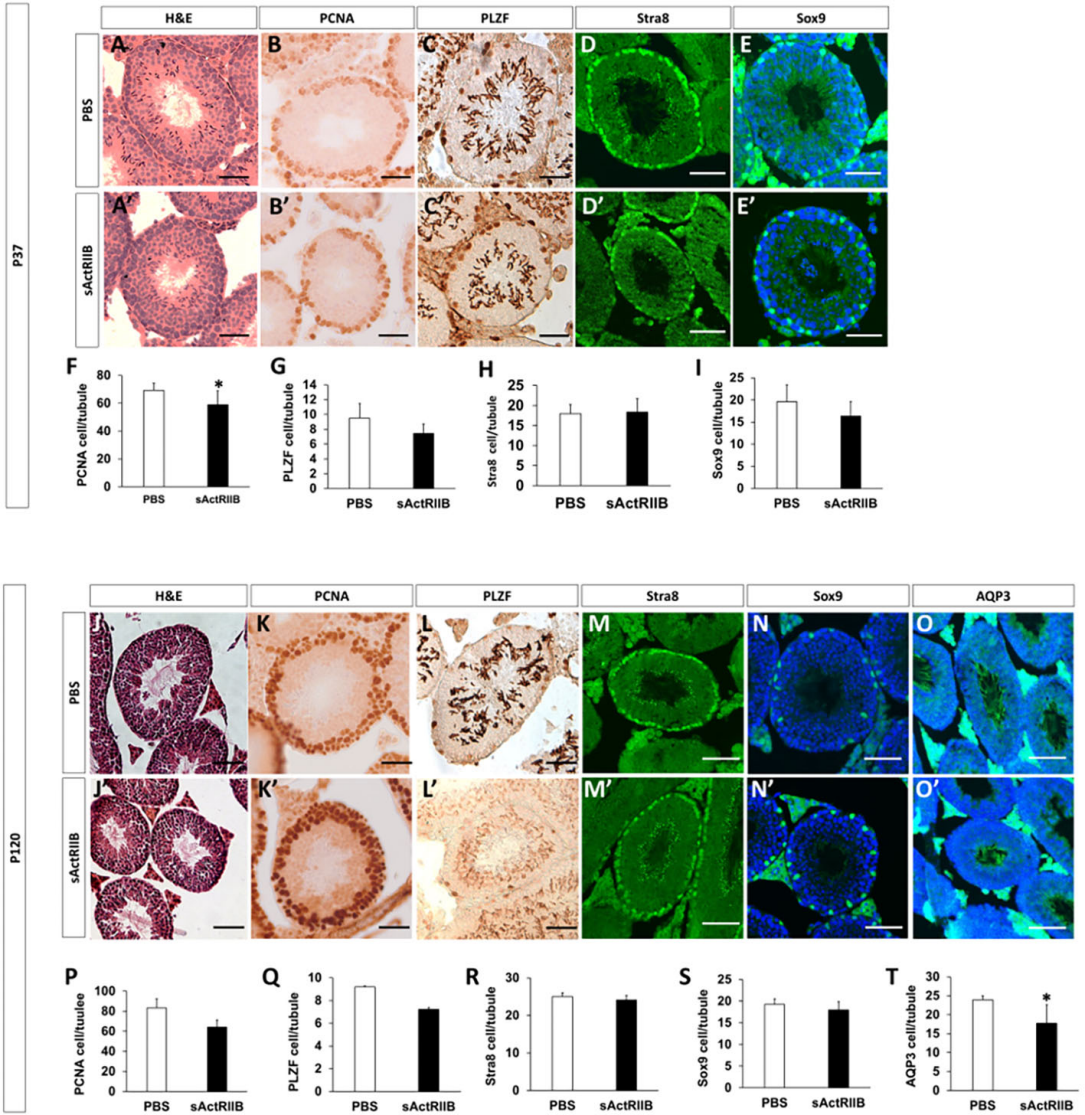
**Histological and immunocytochemical analysis of testis.** (A-I) Tissue from P37 mice. (A,A′) H&E staining of seminiferous tubules in PBS- (A) and sActRIIB-treated (A′) mice, showing smaller tubules in the sActRIIB-treated mice. (B,B′) PCNA-positive cells in the tubules of PBS- (B) and sActRIIB-treated (B′) mice, showing a reduction in PCNA-positive cells in the sActRIIB-treated mice. (C,C′) PLZF-positive cells in the tubules of PBS- (C) and sActRIIB-treated (C′) mice. (D,D′) Stra8-positive cells in the tubules of PBS- (D) and sActRIIB-treated (D′) mice. (E,E′) Sox9-positive cells in the tubules of PBS- (E) and sActRIIB-treated (E′) mice. (F) Quantification of PCNA-positive cells per tubule. (G) Quantification of PLZF-positive cells per tubule. (H) Quantification of Stra8-positive cells per tubule. (I) Quantification of Sox9-positive cells per tubule. *n*=7 PBS-treated male mice, *n*=7 sActRIIB-treated male mice. (J-T) Tissue from P120 mice. (J,J′) H&E staining of seminiferous tubules in PBS- (J) and sActRIIB-treated (J′) mice, showing smaller tubules in the sActRIIB-treated mice. (K,K′) PCNA-positive cells in the tubules of PBS- (K) and sActRIIB-treated (K′) mice, showing a reduction in PCNA-positive cells in the sActRIIB-treated mice. (L,L′) PLZF-positive cells in the tubules of PBS- (L) and sActRIIB-treated (L′) mice. (M,M′) Stra8-positive cells in the tubules of PBS- (M) and sActRIIB-treated (M′) mice. (N,N′) Sox9-positive cells in the tubules of PBS- (N) and sActRIIB-treated (N′) mice. (O,O′) AQP3-positive cells in the tubules of PBS- (O) and sActRIIB-treated (O′) mice. (P) Quantification of PCNA-positive cells per tubule. (Q) Quantification of PLZF-positive cells per tubule. (R) Quantification of Stra8-positive cells per tubule. (R) Quantification of Sox9-positive cells per tubule. (T) Quantification of AQP3-positive tubules as a percentage of total tubules. *n*=4 PBS-treated male mice, *n*=4 sActRIIB-treated male mice. Scale bars: 50 µm. Unpaired Student's *t*-test was used to determine statistical significance. *P<0.05. H&E, Haemotoxylin and Eosin; PCNA, proliferating cell nuclear antigen; PLZF, promyelocytic leukemia zinc finger; Sox9, SRY-box 9; Stra8, stimulated by retinoic acid 8.

The bioinformatic analysis of gene expression demonstrated that key cellular processes and pathways were affected by sActRIIB treatment. We next examined the testes to determine whether these RNA measurements affected cellular processes that are evident at the tissue level. We first assessed cell proliferation in the testes, by quantifying the number of proliferating cell nuclear antigen (PCNA)-expressing cells. Our studies showed that proliferating cells were located adjacent to the basement membrane in the control and sActRIIB-treated samples at both time points (Fig. 6). However, there was a reduction in the number of proliferating cells at P37 but not at P120 (Fig. 6F,P). We next counted the number of spermatogonial stem cells (SSCs) as they give rise to proliferating cells, A and B type spermatogonial cells. To this end, we counted the number of PLZF (also known as ZBTB16)-positive cells per tubule in control and sActRIIB-treated samples. However, we failed to detect any impact of sActRIIB treatment on this parameter at either time point (Fig. 6G,Q). Next, we examined the impact of sActRIIB treatment on the capacity of preleptotene spermatocyte to go into meiosis by assessing Stra8 staining. Again, no difference was found at either time point following sActRIIB treatment (Fig. 6H,R). Thereafer, we counted the number of Sertoli cells, recognised by Sox9 expression, and found no differences in this parameter among the sActRIIB-treated group and controls (Fig. 6I,S). Counting of mature sperm is not possible at P37, as at this age the first wave of spermatogenesis is not completed and therefore these cells have not yet developed. However, this is possible at P120. We found that the number of mature sperm in the lumen, as judged by the development of sperm tails, was significantly reduced by sActRIIB treatment at P120 (Fig. 6O,O′,T).

Because pathological changes in the cell cytoskeletal architecture are often associated with testicular dysfunction (Johnson, 2014), a bioinformatic analysis of gene expression associated with the cytoskeleton was performed. A global heat map of the respective gene expression between the groups revealed strong effects of sActRIIB treatment at P120, with consistent levels of gene expression between the individual animals in the group (Fig. S4). Notably, a very similar gene expression pattern was observed between the sActRIIB and control groups at P37. A more detailed analysis of genes involved in microtubule cytoskeleton organisation revealed a very similar expression pattern, with strong differences between the sActRIIB-treated and control group at P120, and a relatively homogenous expression pattern if the sActRIIB-treated and the control group were compared at P37. Similarly, subsequent analysis of genes involved in microtubule polymerisation and depolymerisation, actin-mediated cell contraction and actin-myosin filament sliding showed strong effects of sActRIIB at P120 (Fig. S5), suggesting that the treatment affects cytoskeletal organisation and function.

**Fig. 7.**
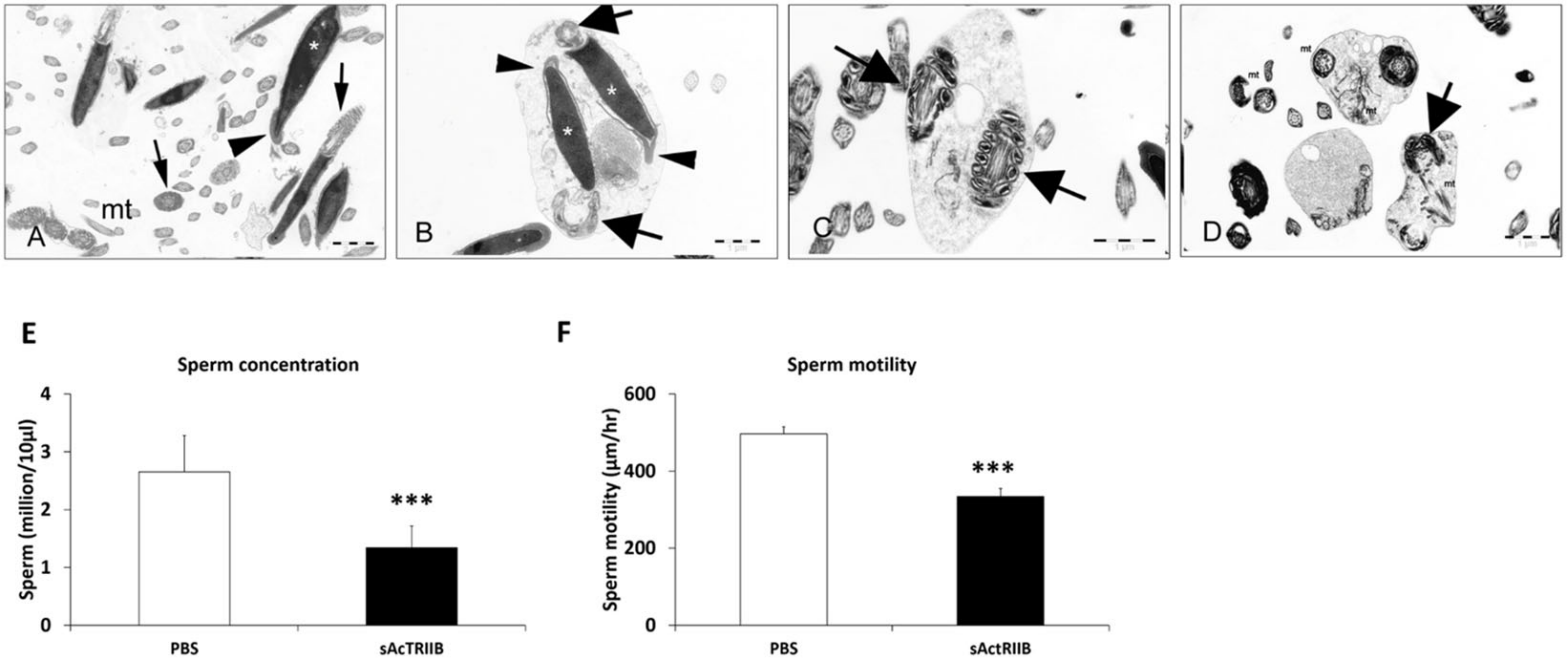
**sActRIIB treatment disrupts spermatozoa morphology, sperm concentration and sperm speed when administered to CD1 mice from P17 until P120.** (A) Transmission electron microscopy of sperm revealed normal spermatozoa morphology in mice treated with vehicle. Acrosome (arrowhead), nuclei (asterisk), midpiece with mitochondrial sheath (arrows), and numerous longitudinal and cross sections of the principal and end pieces showing regular arrangement of microtubules (mt) and outer dense filaments, are shown. (B-D) In the sActRIIB group, few intact spermatozoa were detectable. Instead, a lot of cell debris and displastic sperm was detectable (asterisks, B). This included immature, round sperm containing doubled nuclei (arrowheads, B), with normal acrosomes (arrows, C) and doubled midpieces (arrows, B,D). Spermatozoa also displayed degenerating mitochondria (mt, D) and disarranged microtubules (D) and outer dense filaments. Scale bars: 1 µm. (E) Sperm concentration at 17 weeks of age. (F) Sperm speeds at 17 weeks of age. *n*=6 PBS-treated male CD1 mice, *n*=6 sActRIIB-treated male CD1 mice. Unpaired Student's *t*-test was used to determine statistical significance. ***P<0.001.

The bioinformatic analysis predicted changes in microtubule function and structure. These molecules are key in the development of normal functional sperm. We examined the ultrastructure of sperm, as well as their activity, to determine whether bioinformatic predictions were translated into a biological setting. Hence, we isolated sperm from the epididymis of P120 mice. Transmission electron microscopy images showed sperm from control mice to have normal morphology, i.e. acrosome, large nuclei and sheets of mitochondria (Fig. 7A). In contrast, the sperm from sActRIIB-treated mice showed numerous abnormalities, including many immature sperm, sperm with two or more nuclei, and doubled mid-pieces containing two 9+2 microtubule arrangements (Fig. 7B-D). The number of sperm recovered from the epididymis of sActRIIB-treated mice was significantly lower than that from control mice (Fig. 7E). We also tracked sperm using time-lapse microscopy. Analysis of the video captures revealed a deficit in sperm motility from sActRIIB-treated mice compared to controls (Fig. 7F).

The bioinformatic analysis also predicted changes in cell survival and death. We investigated this further by profiling the expression of cleaved poly (ADP-ribose) polymerase-1 (PARP-1), a marker of cell death. Although this marker was present in the testis, its abundance was not changed as a consequence of sActRIIB treatment (Fig. S3A-C).

**Fig. 8.**
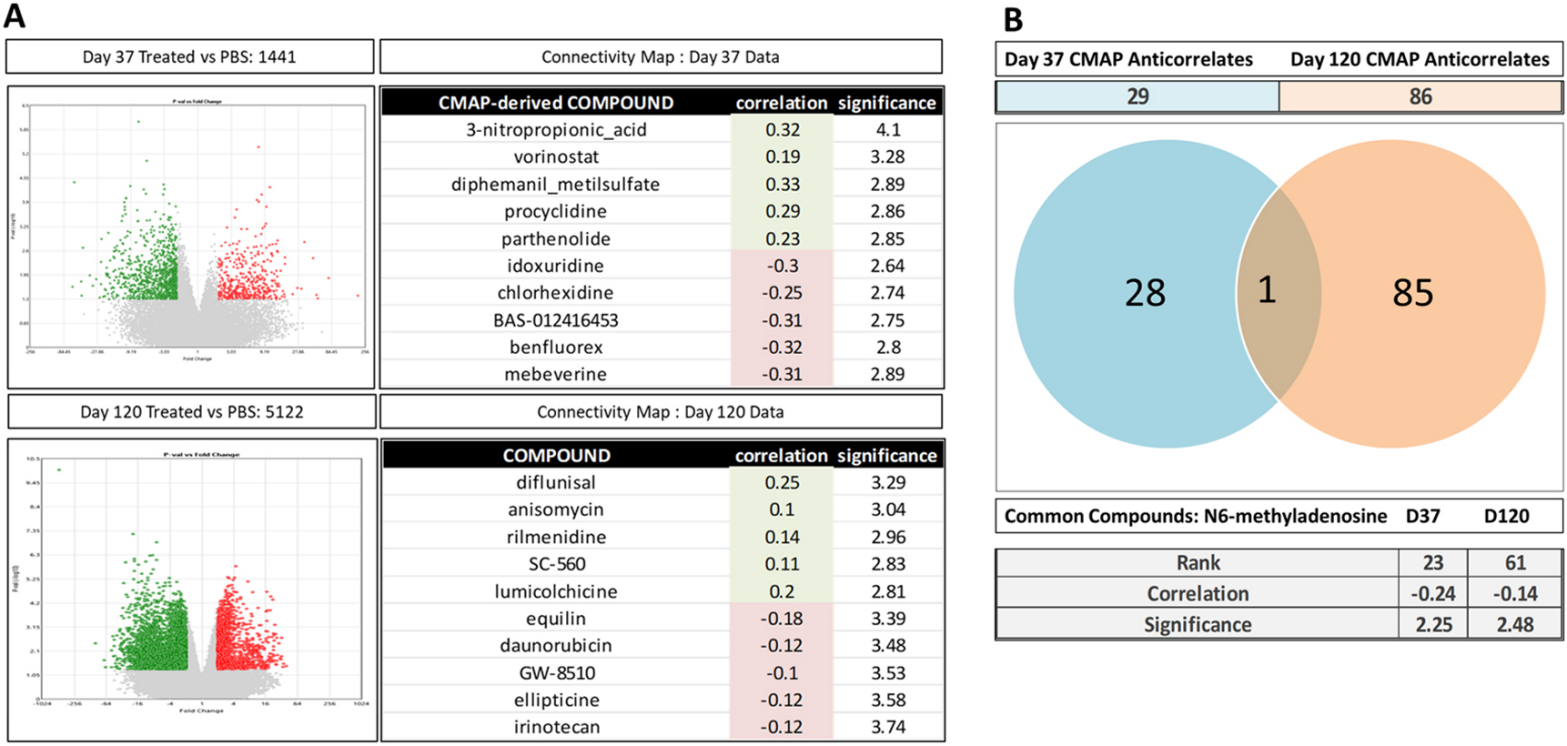
**Connectivity Map (CMAP)-based drug identification.** (A) Differential gene expression analysis between P37 and P120 conditions are represented as volcano plots (*P*>0.01, fold change >±2) and top five correlate and anti-correlate CMAP-derived drug matches at each time point. (B) Venn diagram and CMAP scores of the unique and common drug compound between P37 and P120.

To determine the cohort of CMAP-derived repurposed compounds that could potentially antagonise the phenotypic effects of the treatments described herein, we first assayed the set of genes regulated by each treatment at P37 and P120 compared to the vehicle control (PBS). These investigations identified 1441 genes that were statistically significantly regulated [435 upregulated and 1006 downregulated (treatment versus control *P*<0.05; fold change, −2 or >2)] at P37. Using a similar approach on a set of 5122 genes, several genes were found to be regulated significantly by the treatment at P120 compared to the PBS vehicle control [1574 upregulated and 3548 downregulated (treatment versus control *P*<0.05; fold change, −2 or >2)]. These data demonstrate that, at both P37 and P120, there is a substantial and robust gene expression response (Fig. 8A). We next sought to determine the potential set of US Food and Drug Administration (FDA)-approved compounds that either correlated or anti-correlated with the collection of genes differentially expressed at each of these time points using a CMAP approach. Briefly, genes differentially expressed at P37 or P120 were ranked based on their fold change (i.e. treatment versus PBS control signatures) prior to surveying the CMAP Drug Profile Database as described in Williams et al. (2019). These interrogations revealed a compendium of FDA-approved drugs that either positively or negatively correlated with the P37 and P120 biological signatures. The top five sets of drugs, based on the magnitude of positive and negative correlation, are shown in Fig. 8A. We were primarily interested in identifying the set of drugs with the potential of ameliorating the impact of the treatment described here (i.e. those drugs for which CMAP gene expression profiles anti-correlate with the biological signatures). Accordingly, the top 25 compounds were ranked by the magnitude of anti-correlation and associated significance, described in Table S2 (Williams et al., 2019). Analysis of these data revealed that 29 compounds showed anti-correlating profiles at P37 compared to 86 at P120 (Fig. 8B). We next sought to determine whether any of the compounds were able to ‘antagonise’ the changes in the treatment-driven gene expression at both P37 and P120; these investigations yielded only one compound, *N*^6^-methyladenosine, with ‘anti-correlative’ features, at both time points (Fig. 8B).

To summarise, our findings indicate that the treatment described here induces large-scale genome-wide gene expression differences at both P37 and P120 following initial exposure. Furthermore, we have applied CMAP-based genomic drug repurposing to identify a set of FDA-approved candidate compounds that potentially have the capacity to restore the phenotypic impact of the treatment. Interestingly, our data provide evidence that *N*^6^-methyladenosine has the capacity to ameliorate the dysregulation of genes driven by the treatment at both P37 and P120, and thus represents a strong candidate for future drug-based interventional approaches.

## DISCUSSION

Manipulating the activin/myostatin signalling pathway to promote muscle growth has received much attention in the past decade as our understanding of this signalling cascade has increased, which has inevitably led to the identification of therapeutic targets. We and others have developed readily injectable molecules that attenuate signalling through one of the key cell surface receptors, ActRIIB, which plays a major role in activin/myostatin signal transduction. These molecules all promote rapid muscle growth (Alyodawi et al., 2019; Omairi et al., 2016; Rooks et al., 2017). However, we and others have documented that these interventions can also result in unwanted side effects (Campbell et al., 2017). In particular, we have focused, in a number of studies, on the detrimental effect of sActRIIB on testis development that accompanies muscle growth (Vaughan et al., 2020a,b). Here, we examine the molecular basis underpinning the impact of sActRIIB on the testis.

We found that short-term treatment had a positive impact on muscle growth but was accompanied by a stunting of testis development, a result in keeping with our previous findings (Vaughan et al., 2020b). However, following the cessation of sActRIIB treatment, the muscle growth phenotype was lost but the detrimental impact on testis size remained. The temporal profile of muscle growth following the withdrawal of sActRIIB is interesting; both treated and control muscle weigh the same at P120 despite the increased size of sActRIIB-treated muscle at P37, right after the time when the treatment was stopped. The key feature here is that in the control animals there was a phase of muscle growth, which was not observed in the animals that had been treated with sActRIIB. It is possible that pre-treatment with sActRIIB either prevents the muscle growth program or that the muscle is refractory to molecules that promote myogenic hypertrophy. Alternatively, both cohorts may display the same amount of muscle as a consequence of experiencing identical load following the cessation of treatment. Future studies will explore these and other possible reasons for the muscle phenotype.

A key issue that needs to be addressed concerns the development of side-effects following the use of sActRIIB (or similar-acting molecules). We are not the first to report adverse outcomes of their use (Campbell et al., 2017), but nevertheless it is interesting that others have not encountered such issues. This could simply be that the focus of such studies has been on muscle, and side-effects have been overlooked. However, it is also worth considering the context in which we report the side-effects of sActRIIB. Here, we have used a model to promote muscle growth in a healthy mouse, in which the levels of muscle atrophy-inducing molecules are low when high levels of sActRIIB have been administered. In such a scenario, we would essentially neutralise all activin/myostatin. This scenario could be very different in the setting of a muscle-wasting condition, when lower levels of sActRIIB are used. We note that others have used a similar molecule at the same concentrations but in a context of muscle wasting without noting adverse effects on the testis (Hawinkels et al., 2016). It is possible that, in a model of muscle wasting, activin/myostatin levels are high and the dose of sActRIIB used is stoichiometrically unable to completely neutralise high amounts of the ligands, hence the adverse effect on the testis is not seen. We suggest that, in the latter condition, the lower levels of activin/myostatin may be sufficient for testis development but still capable of preventing muscle atrophy. Again, future work is planned to test this line of thinking.

We have explored the long-term impact on the testis of a limited exposure to sActRIIB in considerable detail, both ranging from studies of organ structure and function down to in-depth molecular analysis. One key question we aimed to address was the nature of the mechanisms underpinning the testis phenotype at the two time points, specifically whether the molecular mechanisms initiated by sActRIIB remained the same over time or whether they evolved. The protein expression data were our first indication that the phenotype evolved; initially there was a decrease in testicular pSMAD2 levels induced by sActRIIB but later these were normalised. However, a converse effect was detected when we profiled levels of pERK. It is well established that several signalling pathways can be activated through the ALK receptors (Massy and Drueke, 2016). Interestingly, our work shows that the ligand trap does not induce activation of the AKT pathway in testis, which contrasts with the situation in muscle, where the activation of this pathway underpins the development of muscle hypertrophy. It has been postulated that the identity of the activated cascade may be determined by ligand concentration or by the presence of specific co-receptors, as well as other factors. Irrespective of what is driving signalling in the tissues, we can conclude that there are changes in signalling activity over time.

The gene expression data have revealed another key feature underpinning the testis phenotype. We show that the number of differentially expressed genes increases greatly with time. There were 257 genes that showed greater than 2-fold difference at the early time point, whereas this number had risen to over 1800 by P120. This type of amplification of a molecular phenotype has previously been noted in a number of other studies [e.g. during ageing (Bryois et al., 2017)], and these studies have shown that the deregulation of a relatively small number of genes initiates large-scale changes owing to their impact on the expression of key molecules including transcription factors, regulators of epigenetic modifiers and genomic stability. Although the number of changes in gene expression increased greatly over time, we suggest that the actual number could be even higher as we have profiled the entire tissue. Herein, it is highly likely that cell-specific changes could be masked by one gene being upregulated in a certain cell type while it is downregulated in another, leading to the erroneous conclusion that it has not changed. This issue is highlighted by the bioinformatic analysis of genes associated with cell proliferation. Here, we showed that that there was a major difference in the profile of transcripts associated with this cellular activity at the late time point. However, there was no change in the number of cells in the cell type that was the one of the most mitotically active (SSCs). Future studies are planned using single-cell transcriptomics, which will not only circumvent this problem but will ultimately identify the cell types affected by sActRIIB. Nevertheless, our work shows that a brief exposure to sActRIIB initiates a cascade of secondary changes.

The bioinformatic analysis made predictions related to the expression of genes associated with cytoskeletal function, in particular microtubule organisation following a systemic treatment with sActRIIB. These predictions were borne out by our studies profiling the structure and function of the sperm. One of the most prominent features of sperm is the 9+2 microtubular organisation in the mid-piece as well as the tail (Lehti and Sironen, 2017). Abnormal sperm flagella have been associated with male infertility in a number of conditions, in particular Kartagener syndrome (Afzelius, 1976). We found that sActRIIB induced numerous changes to the structure and function of sperm. At the ultrastructural level, we noted cells that lacked the canonical 9+2 microtubule organisation or tails that lacked these structures. Importantly we showed that there was a defect in their function; sperm from sActRIIB-treated mice showed lower motility. However, it is important to note that microtubules play an important role not only in sperm motility but also during their development. Microtubules play key roles in the function of Sertoli cell structure and function (Amlani and Vogl, 1988; Vogl, 1988), in the spermatogonia (Vogl et al., 1995) and during their development (O'Donnell and O'Bryan, 2014). Therefore, the deregulation of the microtubular system is likely to have a major impact on the development of the testes.

Bioinformatic analysis of the testis transcriptome identified not only genes that were associated with microtubule organisation but also those that have a major role in sperm development and function. Two of the most highly upregulated microtubule-associated genes were *Hspa1a* and *Cib1*. *Hspa1a* has been shown to be a key spermatozoa molecule that is essential for their structural and functional competence (Kumar et al., 2020). Furthermore, *Cib1* has been shown to be essential for mouse spermatogenesis (Yuan et al., 2006). A key question for future studies is how activin/myostatin signalling regulates microtubule gene expression. There is a dearth of information on this subject in the mammalian context. However, there is precedent in *Drosophila* for such a system with a clear link originating from the ligand (Dawdle) to the microtubule components (Ellis et al., 2010). The latter data could be deployed as a genetic template to investigate the link between activin signalling and microtubular gene expression.

As well as identifying possibly molecular causes of the testis phenotype induced by sActRIIB treatment, we have used the gene expression profiles to identify compounds that may possibly reverse the detrimental impacts of the intervention. Herein, we used the CMAP platform to identify molecules that are predicted to induce gene expression changes that are opposite to those caused by sActRIIB. These include readily available molecules including Benflourex, a drug with possible utility in the treatment of type 2 diabetes (Ravel and Laudignon, 1996), as well as Equilin, which is a component of a hormone replacement therapy (Takei et al., 2016). These drugs were predicted to be effective only at one of the two (P37 and P120) time points examined in this study. We interrogated the data for a drug/molecule that would be effective at both time points, which revealed the name of only one compound: *N*^6^-methyladenosine. This is a novel medicinal compound as it is normally associated with the molecular modification of RNA, which impacts on nuclear transport, pre-mRNA splicing, microRNA biogenesis stability and translation (Zhang et al., 2019). However, there are studies showing that *N*^6^-methyladenosine has direct biological activity, including its ability to regulate the activity of ATP receptors as well as the expression of key enzymes (Ribeiro and Sebastião, 1984; Burke and Joh, 1985). Although the CMAP analysis has identified putative attenuators of the testis phenotype, caution must be exercised in deploying some of the molecules as they may bring about massive disruption to organismal homeostasis [disruption of protein synthesis by the CMAP-identified compound chlorhexidine (Pucher and Daniel, 1992)]. Nevertheless, we believe that it is a good starting position to overcome the side effects of sActRIIB, but one which will need to be refined, especially by using single-cell transcriptomics data.

In summary, we have defined the molecular changes that develop in the testis because of using a regime aimed at promoting muscle growth. We show that short-term use of sActRIIB initiated temporally expanding molecular changes, many of which are predicted to impact on the structure and function of sperm. This projection was further verified in cellular and ultrastructural profiling to reveal abnormalities in sperm number, structure and motility.

## MATERIALS AND METHODS

### Ethical approval

Mouse-based experiments were performed under the auspices of a UK project licence, conforming to the Animals (Scientific Procedures) Act 1986. All procedures were approved by the Animal Care and Ethics Review committee of the University of Reading. Mice were sacrificed through Schedule 1 killing.

### sActRIIB-Fc production

The recombinant fusion protein was produced in house. The ectodomain (ecd) of human sActRIIB was amplified via PCR with the primers 5′-GGACTAGTAACATGACGGCGCCCTGG-3′ and 5′-CCAGATCTGCGGTGGGGGCTGTCGG-3′ from a plasmid containing the human ActRIIB sequence (in pCR-Blunt II-TOPO AM2-G17 ActRIIB, IMAGE clone no. 40005760; The IMAGE Consortium). A human IgG1 Fc domain with a COOH-terminal His6 tag was amplified by PCR (5′-GCAGATCTAATCGAAGGTCGTGGTGATCCCAAATCTTGTGAC-3′ and 5′-TCCCTGTCTCCGGGTAAACACCATCACCATCACCATTGAGCGGCCGCTT-3′) from the pIgPlus expression plasmid. The subcloning of these products was done into the pGEM-T easy (Promega) vectors, sequenced, and fused before cloning into the expression vector pEFIRES-P (Hobbs et al., 1998). For the final protein production, Chinese hamster ovary (CHO) cells were transfected with the above-mentioned ActRIIBecd-FcHis6 expression vector via lipofection (Fugene 6; Roche) and selected with puromycin (Sigma-Aldrich, Lyon, France). During selection, cells were grown in Dulbecco's modified Eagle medium supplemented with 2 mmol/l L-glutamine, 100 μg/ml streptomycin, 100 IU/ml penicillin and 10% fetal calf serum. For large-scale expression, cells were adapted to serum-free CD OptiCHO medium (Gibco) supplemented with 2 mmol/l L-glutamine and grown in suspension in an orbital shaker. Cell culture supernatants were clarified by filtration through a 0.22-µm membrane (Steritop; Millipore). Next, NaCl and imidazole were added, and the solution was pumped through a Ni-loaded HiTrap Chelating column (GE Healthcare Life Sciences-Cytiva, Uppsala, Sweden) at 4°C. Protein was eluted by raising imidazole concentrations, dialysed against PBS, and finally concentrated with an Amicon Ultra concentrator (30000 MWCO; Millipore). The purity of our sActRIIB-Fc preparation after immobilised metal affinity chromatography purification was estimated to be over 90% based on silver-stained sodium dodecyl sulphate–polyacrylamide gel electrophoresis.

### Animal maintenance

C57 strain mice were bred and maintained at the Biological Resources Unit of the University of Reading in normal housing conditions, which included *ad libitum* access to food and water. sActRIIB was administered via the intraperitoneal route starting on P17 on a twice weekly basis at a dose of 10 mg/kg until P35. Mice were then maintained without further sActRIIB treatment until the time of sacrifice (either P37 or P120).

### Histology and immunocytochemistry

Testes were fixed in 4% paraformaldehyde for histology and immunocytochemistry. Thereafter, tissue was processed for microtome sectioning firstly by embedding in paraffin. Then, 8-µm sections were produced, which were treated for antigen retrieval using 10 mM citrate buffer and processed further as described in Vaughan et al. (2020b). Details of primary and secondary antibodies are provided in Table S1.

### Electron microscopy

Epidydimal sperm samples were centrifuged and the resulting pellet embedded in 2% agarose for transmission electron microscopy. Thereafter, cells/tissue samples were contrasted using 1% osmium tetroxide in PBS for 45 min at room temperature and 1% uranyl acetate (45 min at room temperature in 70% ethanol). Agar blocks were dehydrated and embedded in epoxy resin (Durcupan, Sigma-Aldrich, Germany). Ultrathin sections were prepared using a Leica UC6 ultra-microtome and analysed using a Philipps CM100 transmission electron microscope.

### Bioinformatics

Genome-wide gene expression of testes from control mice (PBS) and mice treated with the soluble form of the activin type IIB receptor (sActRIIB) at P37 and P120 was analysed in independent triplicate using Affymetrix GeneChip^®^ Mouse430_2 Arrays. Raw image data were analysed with Affymetrix^®^ Expression ConsoleTM Software, and gene expression intensities were normalised and summarised with the robust multiarray average algorithm (Irizarry et al., 2003). To identify genes differentially expressed between treated and control mice, a comparison analysis using Affymetrix Transcriptome Analysis Console (TAC) 4.0 Software was performed. Gene expression was considered as changed if transcript levels between treated and control groups were differential with a 2-fold change and a *P*-value of <0.05. For the pathway analysis, sets of differentially expressed genes (corresponding to identified transcripts, |Log2FC|≥1, q≤0.05) were assessed by Qiagen’s Ingenuity^®^ Pathway Analysis (IPA^®^; spring release 2019). Specifically, we carried out a canonical pathway, followed by disease and function associations' analysis. *P*-values, which were ascertained by right-tailed Fisher's exact test following Benjamini and Hochberg correction, indicated the robustness of correlation between a subset of differentially expressed genes of the dataset with a given biological function. Annotations demonstrating a |*z*-score| >2.0 were taken into account for the heat-map representation (predicted activation or inhibition of a given biological function), as described by Pezzini et al. (2017). Heat maps were drawn with Heatmapper (Babicki et al., 2016), utilizing Euclidean distance measures with leaf clustering.

### Connectivity mapping

Total RNA was extracted from biological replicates using the Agilent Micro RNA system as per the manufacturer's instructions (Agilent). The integrity of the total RNA from each sample was determined using a 2100 Bioanalyzer system in combination with a RNA pico chip as per the manufacturer's instructions, and RNA quantities were normalised using Nanodrop spectrophotometric measurement. Genome-wide transcriptomic representation from 500 pg total RNA from each sample was determined via Affymetrix MOE430_2 GeneChips (Thermo Fisher Scientific) in combination with Nugen Ovation WTA Pico V2 Library preparation as per the manufacturer's instructions. Differential expression data were quality controlled (e.g. PCA analysis) and analysed using the TACX software package (Thermo Fisher Scientific) as per the manufacturer's guidelines. The microarray data in this study were summarised and normalised using MAS5 pre-processing. Statistically significant differentially expressed gene lists [*P*<0.05; fold change >±2] were submitted for CMAP analysis as described previously (Williams, 2013).

### Statistical analysis

Data are presented as mean±s.e. Student's *t*-test for independent variables was used to identify the differences between two groups. Data were analysed to establish whether they followed a normal distribution pattern. Statistical analysis was performed in GraphPad Prism 5 with statistically significant differences considered at *P*<0.05. All other statistical tools are described in the appropriate Materials and Methods section and in the Results section.

## Supplementary Material

Supplementary information
