# Supplementary material for: A muscle growth-promoting treatment based on the attenuation of activin/myostatin signalling results in long-term testicular abnormalities
Source: Dis Model Mech. 2021 Feb 19;14(2):dmm047555. doi: 10.1242/dmm.047555 (PMC7903914; doi:10.1242/dmm.047555)
Supplement: Supplementary information [file dmm-14-047555-s1.pdf]

Supplementary Figure 1

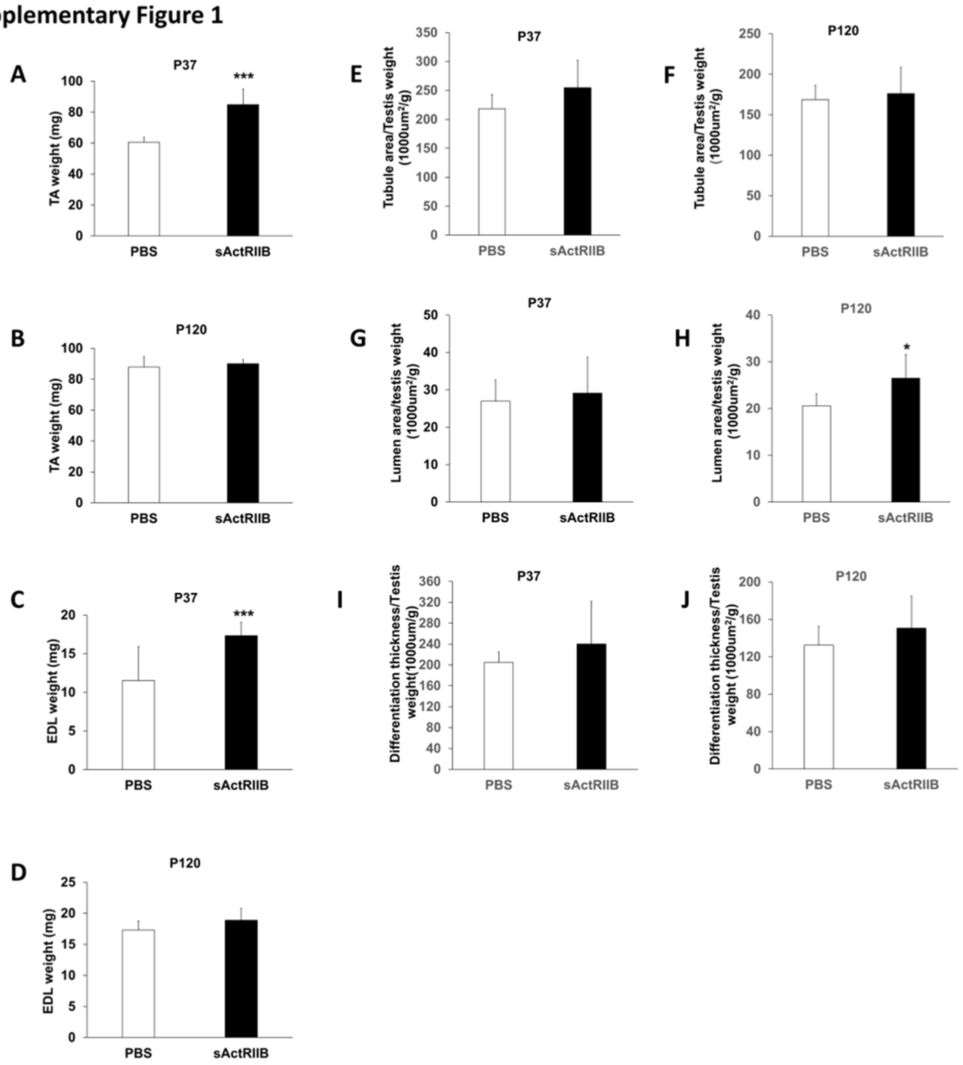

Figure S1. Muscle weights at day 37 and 120 for Tibialis Anterior (A-B) and EDL (C-D). Normalization of tubule area (E-F), lumen area (G-H) and differentiation thickness (I-J) to testis weight. For P37 experiments n=7 PBS treated male mice, n=7 sActRIIB treated male mice. For P120, n=4 PBS treated male mice, n=4 sActRIIB treated male mice. Student's t-Test used for statistical significance. *<p<0.05, ***p<0.001.

Disease Models & Mechanisms • Supplementary information

Supplementary Figure 2

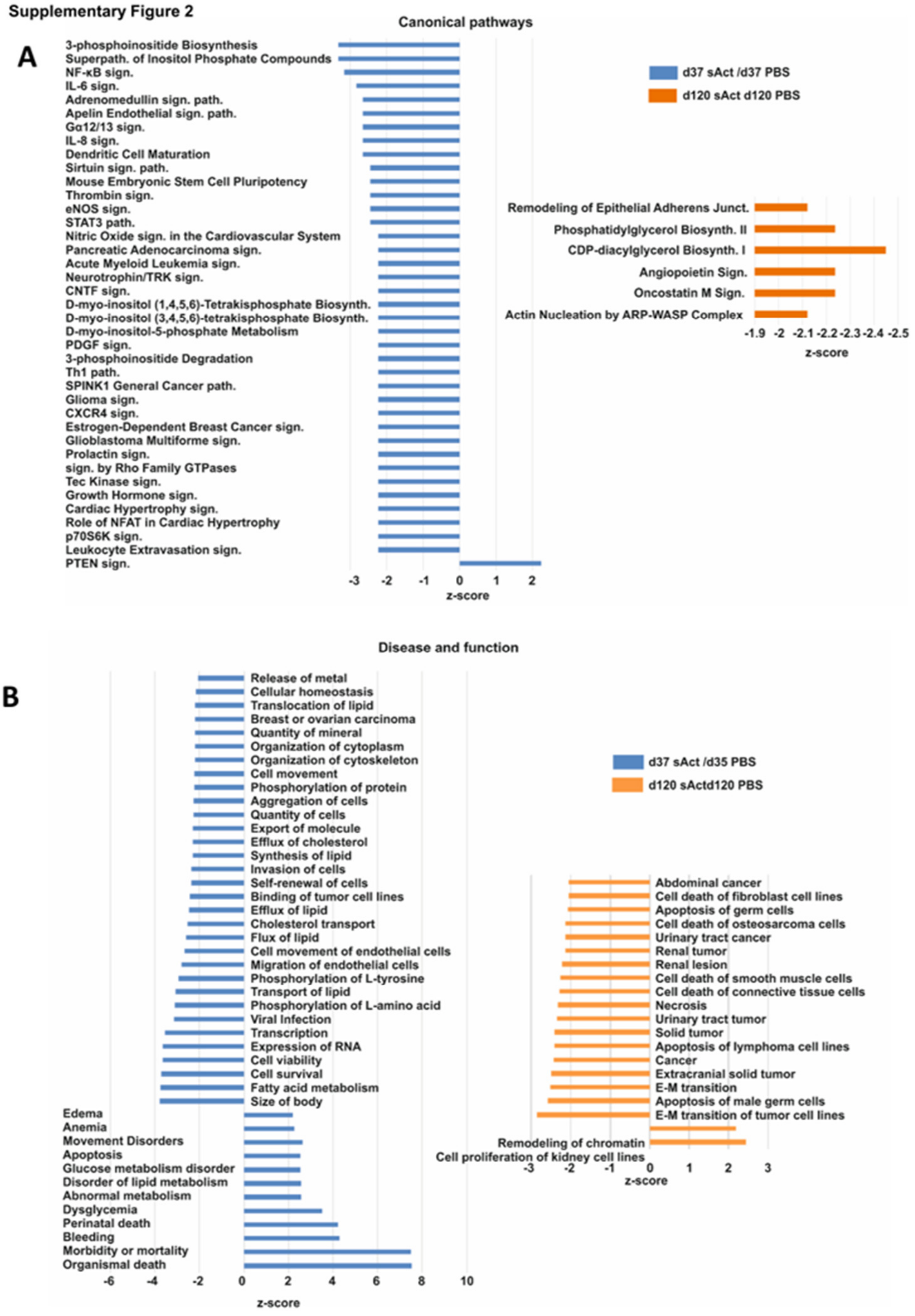

Figure S2. (A) Canonical pathways and (B) disease and associated functions sorting of pathways predicted to be differentially affected by sActRIIB treatment. The |z-score| ≥ 2 was used as a criterion for significant activation or inactivation of a given pathway or process. Here are the changes in canonical pathways and diseases and function indicators following comparison of teste gene expressions patterns for the impact of sActRIIB treatment at the two time points examined.

Disease Models & Mechanisms • Supplementary information

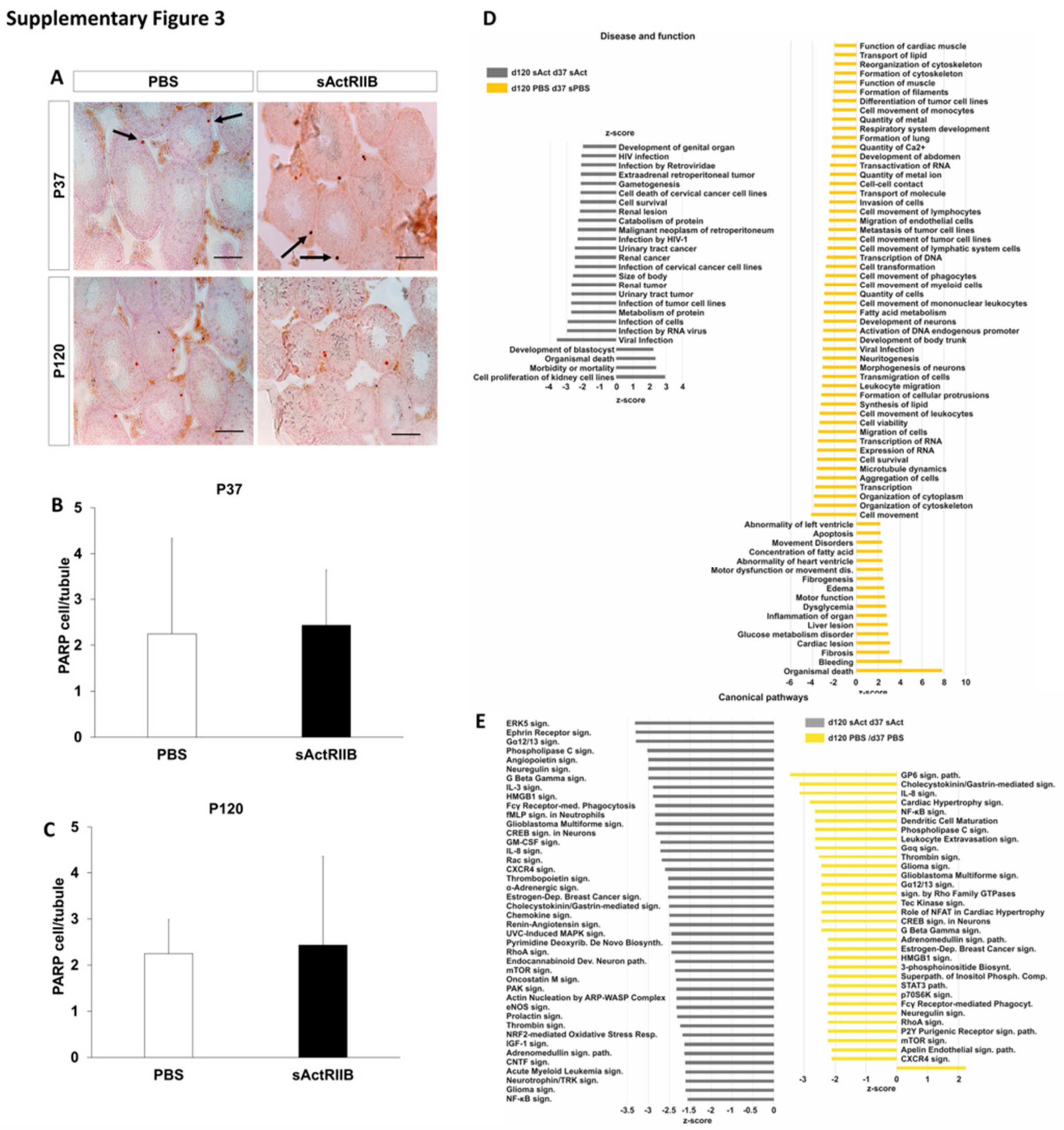

Figure S3. (A) Immunostaining for cleaved PARP as a proxy for apoptosis in the testis (arrows). (B-C) Quantification of cleaved PARP in testis of sActRIIB treated mice. For P37 experiments n=7 PBS treated male mice, n=7 sActRIIB treated male mice. For P120, n=4 PBS treated male mice, n=4 sActRIIB treated male mice. Student's t-Test used for statistical significance. Scale for all images represents 50µm. (D) Canonical pathways and (E) disease and associated functions sorting of pathways predicted to be differentially affected in the indicated experimental conditions. The |z-score| ≥ 2 was used as a criterion for significant activation or inactivation of a given pathway or process.

Disease Models & Mechanisms • Supplementary information

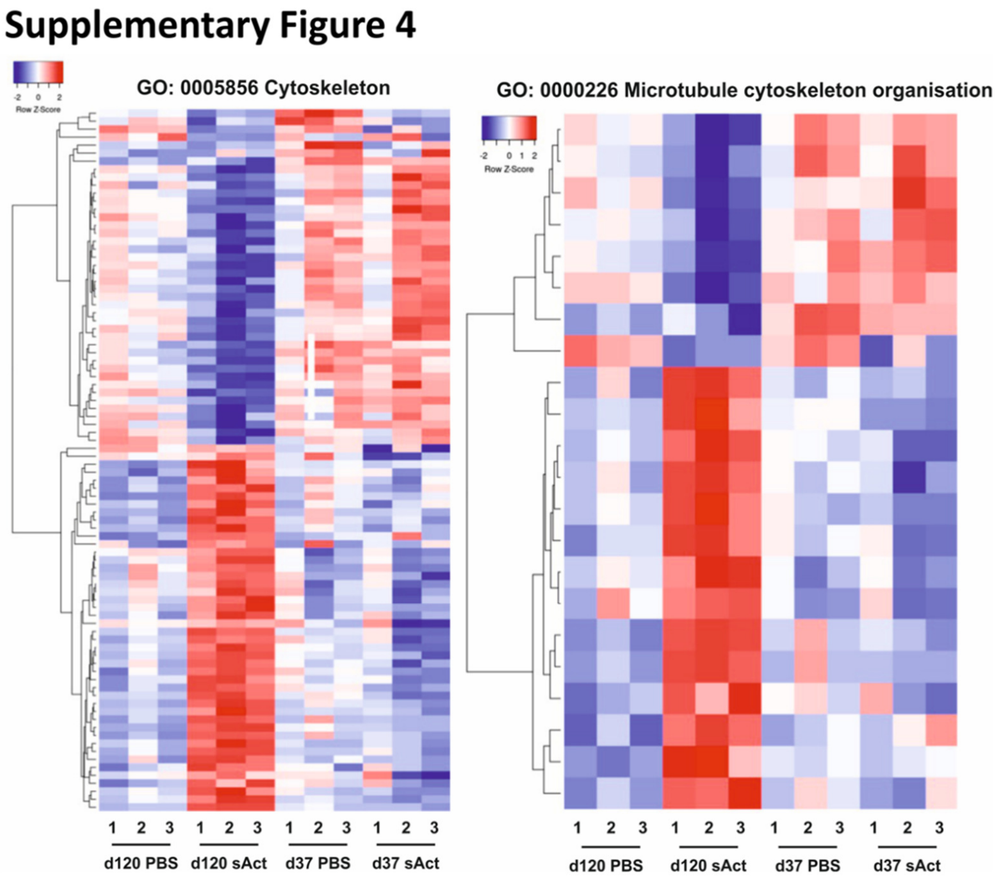

Figure S4. Heat map representations of differentially expressed transcripts within the Gene Ontology Categories `Cytoskeleton`, and `Microtubule cytoskeleton organisation`. Euclidean distance measures and average leaf clustering was applied to rows. The experimental conditions are indicated on the bottom. Each column represents one individual animal.

Supplementary Figure 5

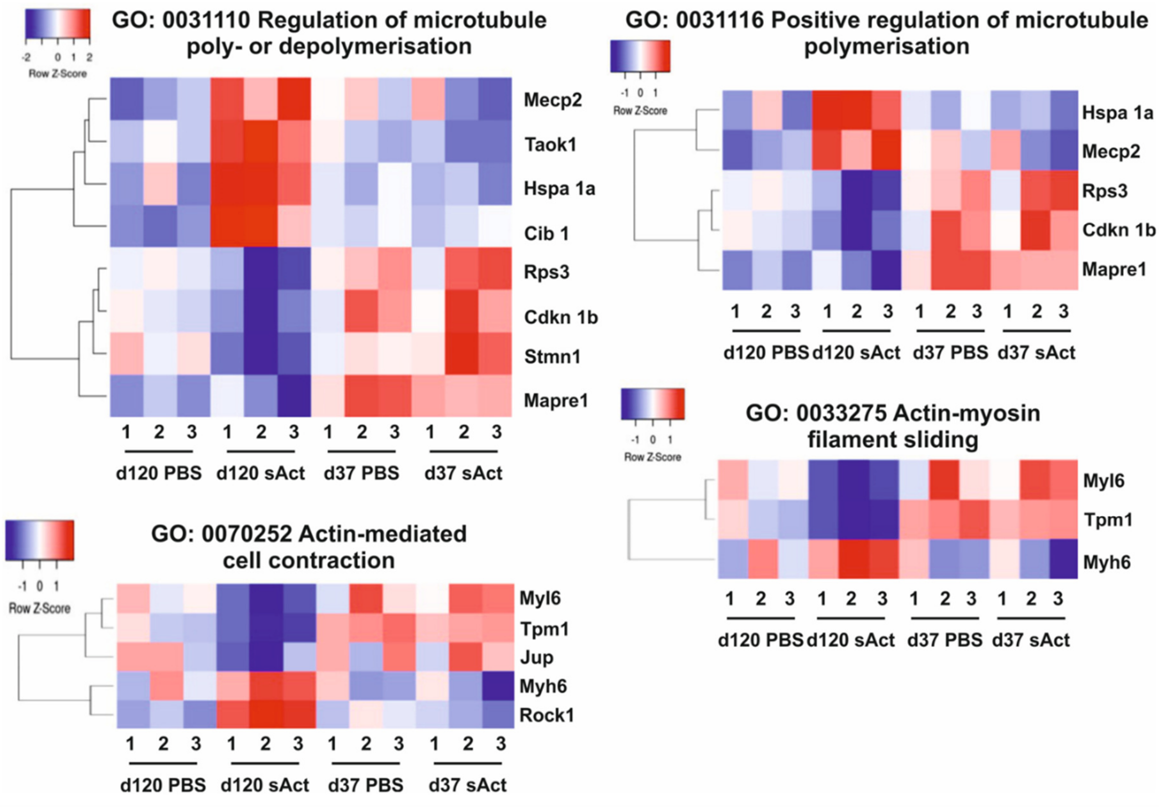

Figure S5. Heat map representations of differentially expressed transcripts within the Gene Ontology Categories `Regulation of microtubule poly- or depolymerization`, `Positive regulation of microtubule polymerization`, `Actin-mediated cell contraction`, and `Actin-myosin filament sliding`. Euclidean distance measures and average leaf clustering was applied to rows. The experimental conditions are indicated on the bottom. Each column represents one individual animal.

Disease Models & Mechanisms • Supplementary information

Table S1. Antibody details

**Primary antibodies**<br>**Antigen**	**Species**	**Dilution**	**Supplier**
PCNA	Mouse	1:200	Cell signalling Technology #25865
PLZF (D-9)	Mouse	1:250	Santa Cruz Biotechnology #sc-28319
Stra8	Rabbit	1:1000	Abcam ab49405
Sox9	Rabbit	1:1000	Abcam ab185966
AQP3	Rabbit	1:800	Abcam ab125219
Phospho-Smad2 (Ser465/467)/Smad3 (Ser423/425)	Rabbit	1:1000	Cell signalling Technology #8828
Smad2/Smad3	Rabbit	1:1000	Cell signalling Technology #3102
Phospho-AKT (Ser473)	Rabbit	1:1000	Cell signalling Technology #4060
AKT	Rabbit	1:1000	Cell signalling Technology #9272
Phospho-p44/42 MAPK (Erk1/2) (Thr202/Tyr204)	Rabbit	1:1000	Cell signalling Technology #9101
p44/42 MAPK (Erk1/2)	Rabbit	1:1000	Cell signalling Technology #4695
Alpha-tubulin	Mouse	1:1000	Cell signalling Technology #3873s
PARP	Rabbit	1:100	Abcam ab32064

**Secondary antibodies**	**Species**	**Dilution**	**Supplier**
AlexaFluor 488 anti-mouse	Goat	1:200	Invitrogen
Rabbit anti mouse IgG HRP	Rabbit	1:200	DAKO #PO260
Goat anti-rabbit IgG HRP	Goat	1:5000	ThermoFisher scientific #65-6120

Table S2. The top 25 (as determined by cumulative correlation and significance score) anti-corelating CMAP-derived drugs for both Day 37 and 120 samples.

Rank	Day 37 CMAP-derived compound	Correlation	Significance	Day 120 CMAP-derived compound	Correlation	Significance
1	mebeverine	-0.31	2.89	irinotecan	-0.12	3.74
2	benfluorex	-0.32	2.8	ellipticine	-0.12	3.58
3	BAS-012416453	-0.31	2.75	GW-8510	-0.1	3.53
4	chlorhexidine	-0.25	2.74	daunorubicin	-0.12	3.48
5	idoxuridine	-0.3	2.64	equilin	-0.18	3.39
6	perhexiline	-0.25	2.59	estriol	-0.12	3.35
7	NU-1025	-0.24	2.56	flufenamic_acid	-0.16	3.33
8	cefotiam	-0.21	2.49	cloperastine	-0.12	3.28
9	Prestwick-981	-0.23	2.49	clemizole	-0.13	3.28
10	mianserin	-0.27	2.46	propafenone	-0.13	3.27
11	saquinavir	-0.31	2.44	benzonatate	-0.2	3.27
12	foliosidine	-0.3	2.42	carbachol	-0.12	3.25
13	hexylcaine	-0.32	2.41	picotamide	-0.19	3.24
14	wortmannin	-0.16	2.37	Prestwick-967	-0.23	3.23
15	fursultiamine	-0.37	2.35	sodium_phenylbutyrate	-0.17	3.23
16	clemastine	-0.19	2.35	pramocaine	-0.22	3.21
17	Prestwick-1082	-0.17	2.34	fluorometholone	-0.13	3.18
18	niclosamide	-0.22	2.33	camptothecin	-0.09	3.15
19	fluorocurarine	-0.24	2.3	valproic_acid	-0.1	3.15
20	etilefrine	-0.3	2.29	xylometazoline	-0.14	3.12
21	5252917	-0.19	2.29	rofecoxib	-0.21	3.09
22	hydroxyzine	-0.35	2.26	cortisone	-0.12	3
23	N6-methyladenosine	-0.24	2.25	resveratrol	-0.11	2.97
24	lisinopril	-0.19	2.23	hydrastine_hydrochloride	-0.15	2.96
25	ifosfamide	-0.27	2.22	6-azathymine	-0.11	2.96

Disease Models & Mechanisms • Supplementary information
